# Impact on Postoperative Pain and Recovery of a Regional Analgesia Strategy Based on the Surgical Approach for Lung Resection: A Prospective Observational Study

**DOI:** 10.3390/jcm11051376

**Published:** 2022-03-02

**Authors:** Marion Trouillard, William Dupuis, Hélène Siaudeau, Florian Denou, Emmanuelle Longeau, Maxime Léger, Myriam Ammi, Cyril Sargentini, Sigismond Lasocki, Emmanuel Rineau

**Affiliations:** 1 Department of Anesthesiology and Intensive Care, University Hospital of Angers, 49100 Angers, France; marion.trd@gmail.com (M.T.); williamj.dupuis@hotmail.fr (W.D.); helene.siaudeau@chu-angers.fr (H.S.); florian.denou@hotmail.fr (F.D.); emmanuelle.longeau@chu-angers.fr (E.L.); maxime.leger@chu-angers.fr (M.L.); cysargentini@chu-angers.fr (C.S.); silasocki@chu-angers.fr (S.L.); 2 Department of Cardiovascular and Thoracic Surgery, University Hospital of Angers, 49100 Angers, France; myriam.ammi@chu-angers.fr

**Keywords:** postoperative pain, postoperative recovery, epidural analgesia, intrathecal analgesia, erector spinae plane block, lung surgery, video-assisted thoracic surgery, thoracotomy

## Abstract

Various regional anesthesia (RA) techniques were shown to reduce pain after lung surgery, but controversies remain regarding the best technique to use to improve recovery. In this observational prospective study, the aim was to assess the efficacy of an RA strategy depending on the surgical approach. Patients who underwent lung surgery were included if an RA was planned following our unit procedure (erector spinae plane block (ESP) for video-assisted thoracic surgery (VATS) and thoracic epidural analgesia (TEA) or intrathecal analgesia (IA) for thoracotomy). Patients were compared according to the RA used. In total, 116 patients were included, 70 (60%), 32 (28%), 14 (12%) in the ESP, TEA and IA groups, respectively. Between Day 1 and Day 3, median NRS values were ≤4 at rest, and <50% patients experienced moderate-to-severe pain in each group. There were no significant differences in opioid consumption and in pain at rest or during chest physiotherapy on Days 1 and 2 between groups. However, patients who received an IA had lower NRS than other groups on Day 0 and 3 and a shorter length of hospital stay in comparison with those who received a TEA. Thus, in our institution, a strategy combining ESP for VATS and TEA, or IA for thoracotomy, allowed for effective analgesia after a lung resection. Interestingly, IA appeared to be more effective than TEA in reducing the length of hospital stay and pain on Day 0 and 3.

## 1. Introduction

Lung resection surgery is responsible for major postoperative pain [1], which increases both morbidity and mortality [2]. This pain has a strong impact on patient recovery and increases the length of hospital stay [3]. Regional anesthesia (RA) has a predominant role among pain relief therapies available in this context [4], as it provides strong analgesia and allows morphine consumption and morphine-related side effects to be reduced [5].

Thoracic epidural analgesia (TEA) has long been considered as the preferred technique of analgesia after thoracic surgery as it reduces postoperative pain after video-assisted thoracoscopic surgery (VATS) or thoracotomy, and reduces postoperative ileus [6,7,8,9]. However, epidural analgesia induces a sympathetic block that can cause intra- and postoperative hypotension and acute urinary retention, and catheter placement can lead to neurological damage in rare cases [10,11]. Thus, various other RA techniques have been developed and assessed in thoracic surgery, such as the paravertebral block, the erector spinae plane block (ESP), and intrathecal analgesia (IA) [12].

The paravertebral block has proved its analgesic efficiency after thoracic surgery and its ability to reduce hypotension, acute urinary retention, pruritus and postoperative nausea and vomiting (PONV) in comparison with TEA [13,14]. Its benefit in reducing postoperative pain has now been shown in both VATS and thoracotomy surgeries [15,16]. Therefore, the recent 2019 guidelines from the French Societies of Cardio-Vascular and Thoracic Surgery (SFCTCV) and of Anesthesia and Critical Care (SFAR) recommended its first-line use (i.e., before epidural analgesia) to facilitate early recovery after pulmonary lobectomy [17]. However, the ESP, more superficially, seems to have similar properties to the paravertebral block [18], and its realization seems to be easier and faster. Since 2016, the ESP has been increasingly used [19], and its use was shown to provide adequate short- [20] and long-term pain control in thoracic surgery [21]. Nevertheless, few studies so far have compared the ESP with other RA techniques. Finally, morphine IA seems to be little used in lung surgery and has been little studied in this context. However, it has shown to provide effective analgesia [22,23,24] and reduce the length of hospitalization stay compared to multimodal analgesia without RA [25].

ESP, TEA, and IA are commonly used in our institution for thoracic surgery, following a unit procedure. The procedure was developed to provide effective analgesia while facilitating postoperative recovery, depending on the type of surgery. To our knowledge, the three chosen blocks have not been evaluated as part of an overall strategy for the management of patients who undergo lung surgery, including different surgical approaches. The aim of our study was to assess the impact of a strategy using these three regional anesthesia techniques on postoperative recovery after lung resection.

## 2. Materials and Methods

We conducted a prospective observational study at Angers University Hospital in France. The study was approved by an Investigational Review Board (Comité d’Ethique du CHU d’Angers, reference number 2019/97). It was registered in the French National Technologies and Civil Liberties Commission (number: ar19-0061v0) and in the ClinicalTrials registry (number: NCT04147754). Patients were informed during anesthesia consultation, and we obtained a patient agreement before inclusion to record their data.

### 2.1. Population

Inclusion criteria were adult patients undergoing an elective lung resection between 1 November 2019 and 1 November 2020 and who had a pre- or intra-operative regional anesthesia technique using either erector spinae plane block, thoracic epidural analgesia or intrathecal analgesia. Non-inclusion criteria were emergency or revision surgery, patients under 18 years of age, pregnancy, patients with legal guardianship, no French-speaking patients or contraindication to regional techniques.

The duration of inclusion period (one year) was chosen in order to obtain a relevant number of patients in relation to the volume of pulmonary surgeries carried out in our unit, while having homogeneous practices in terms of surgery, anesthesia, pain management and postoperative rehabilitation.

### 2.2. Unit Procedure for Analgesic Management

**Figure 1:**
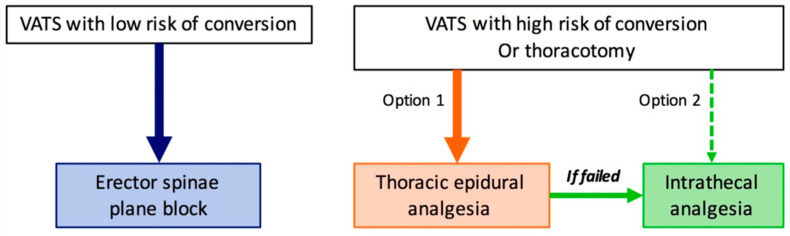
Choice procedure for the regional anesthesia technique to use in lung surgery at Angers University Hospital, France.

In our department, the procedure of choice for the RA technique in lung surgery was based on the surgical approach (VATS or thoracotomy) and the estimated conversion risk to thoracotomy, assessed by the surgeon and discussed with the anesthesiologist (Figure 1). However, the final choice of the RA technique was at the discretion of the anesthesiologist in charge of the patient.

The erector spinae plane block was performed immediately after general anesthesia induction in the lateral decubitus position. A 22-gauge 50 or 80 mm needle (Braun Ultraplex^®^ 360) was inserted at a level between T5 and T8 under in-plane ultrasound guidance. After gentle suction, about 30 mL of 3.8 mg/mL ropivacaine was slowly injected between the erector spinae muscle and its anterior fascia.

The thoracic epidural catheter was inserted on the day of surgery (before induction of anesthesia) or on the day before surgery. In the sitting position, the puncture was made at the T7–T8 interspace with an 18-gauge Tuohy needle (Braun Perifix^®^ ONE). A 2 or 3 mL test dose of 2% xylocaine 0.0005% adrenaline was injected in the epidural space after catheter insertion. At the start of surgery, a bolus (5 to 20 mL) of a 2 mg/mL ropivacaine and 1 µg/mL sufentanil mixture could be carried out in the epidural catheter, followed by an epidural infusion of a 2 mg/mL ropivacaine and 0.5 µg/mL sufentanil mixture. The decision to inject a bolus and the initial epidural infusion rate was chosen by the anesthesiologist in charge of the patient.

Intrathecal analgesia was performed immediately before general anesthesia induction. In the sitting position, a needle (25- or 27-gauge needle depending on the anesthesiologist habit, BD Whitacre^®^) was inserted into the L4–L5 interspace. A single intrathecal bolus was carried out, using about 300 µg of morphine and 25 µg of sufentanil.

After surgery, patients were admitted either to the thoracic surgery ward (after at least one hour of monitoring in the postanesthesia care unit (PACU)) or in the intensive care unit (ICU). The choice was based on patient’s comorbidities, type of surgery (wedge, lobectomy or pneumonectomy, VATS or thoracotomy), intraoperative complications, and the type of RA used. In our unit, patient monitoring was carried out in ICU for patients who had an IA (24 h minimum) and those who had a TEA (as long as the epidural infusion was in progress).

### 2.3. Outcome Measurements

Data were prospectively collected in the preoperative period, during the surgery and in the first three postoperative days. The objectives evaluating the impact of our analgesic strategy were measured in the entire group of included patients, but also in each group of patients who received a different regional analgesia. Thus, three groups of patients were compared: patients who received an erector spinae plane block (ESP group), those who received thoracic epidural analgesia (TEA group), and those who received intrathecal analgesia (IA group).

To assess the efficiency of our analgesic strategy on recovery after lung surgery, our primary outcome was the pain at Day 2 after surgery (at rest and on exertion), using the numerical pain rating scale (NRS) with values between 0 and 10.

Main secondary outcomes included pain at other perioperative times (H2, Days 0, 1, and 3), at rest (morning and evening) and during chest physiotherapy exercises, cumulative morphine consumption until the third postoperative day, morphine-related adverse effects, ICU or hospital length of stay, and effects on pulmonary function (Peak Expiratory Flow (PEF)). Respiratory complications requiring specific therapies (non-invasive ventilation (NIV), high-flow oxygen therapy, re-intubation, new pleural drainage, bronchoscopic suction, lung infection treated with antibiotic therapy), readmissions to ICU, revision surgery requirements and deaths were recorded. The incidence of postoperative neuropathic pain was also assessed.

Data assessing the safety of regional anesthesia were also collected: neurological complications (epidural hematoma, dural breach, motor function impairment, transient radicular irritation, confusion), hemodynamic complications (episodes of fluid bolus requirements or use of vasoactive drugs) and cardiac complications (supra-ventricular tachycardia, acute cardiac failure, cardiac arrest).

As oxycodone and morphine were used, orally and intravenously, an equianalgesic table proposed by the French Society for Palliative Care and Support (available at http://www.sfap.org, accessed on 28 February 2022) was used to obtain the morphine-equivalent consumption, as follows: 1 oral morphine = 1/2 oral oxycodone = 1/3 intravenous (IV) morphine and 1 IV morphine = 1 IV oxycodone.

### 2.4. Data Analysis

Anonymized data were recorded in Excel^®^ software and statistical analysis was performed using JMP^®^ software (SAS Institute, Brie Comte Robert, France).

Data are reported as medians (25–75% interquartiles) or numbers (percentages) for the entire group and for each subgroup of patients. As this study was an observational study aiming to assess the impact of a global strategy on all patients, no sample size calculation and power analysis were carried out prior to inclusions. Numerical data were compared using the Kruskal–Wallis test when a unique *p*-value was given for the overall comparison of the three groups, and a Mann–Whitney test was used when each group was compared with each other. Categorical data were compared using the chi-2 or the Fisher’s exact test. All tests were two-tailed and a *p*-value of less than 0.05 was considered significant.

As pulmonary outcomes are known to be different depending on the surgical approach, and because TEA and IA were mostly carried out in patients who had a thoracotomy incision, a post hoc analysis was performed in the subgroup of patients who had a thoracotomy incision (including converted VATS, lateral, posterolateral and anterolateral thoracotomies), in order to compare outcomes of patients who had a TEA with those who had an IA.

## 3. Results

### 3.1. Population Characteristics

One-hundred and sixteen patients were included: 70 (60%) in the ESP group, 32 (28%) in the TEA group and 14 (12%) in the IA group (flow chart in the Supplementary Material, Figure S1). Patients’ demographic data are detailed in Table 1. The main surgical indication was lung tumor resection (92%). Preoperative spirometry results were not significantly different between the three groups.

### 3.2. Anesthetic and Surgical Data

The unit procedure for the choice of regional anesthesia was followed for 103 (89%) patients, although 3 patients underwent primary thoracotomy in the ESP group, 5 patients underwent VATS only in the TEA group, and 5 patients underwent VATS only in the IA group, without prior high-risk criteria for conversion to thoracotomy (Table 2).

Concentrations and volumes used for the different types of RA followed the unit procedure. For patients in the TEA group, TEA infusion was started intraoperatively (at the beginning of the surgical procedure) for 14 (44%) patients only, and postoperatively for the others. TEA was used with ropivacaine only (i.e., without sufentanil) for eight (25%) patients. The postoperative infusion rate was between 3 and 8 mL/h, with a median rate of 5 (5–6) mL/h.

IV anesthetic data and surgical data are detailed in Table 2. Total doses of remifentanil and propofol were less important in the ESP group. In most cases (69 patients (59%)), the lung resection was a lobectomy.

### 3.3. Postoperative Pain

Using the NRS, the median pain on Day 2 (primary endpoint) for all patients was 2 (0–4.8) in the morning at rest, 0.5 (0–3) in the evening at rest, and 4 (2.5–5) on mobilization, with 46 (40%) patients having at least once a pain score > 3 at rest during this day.

**Figure 2:**
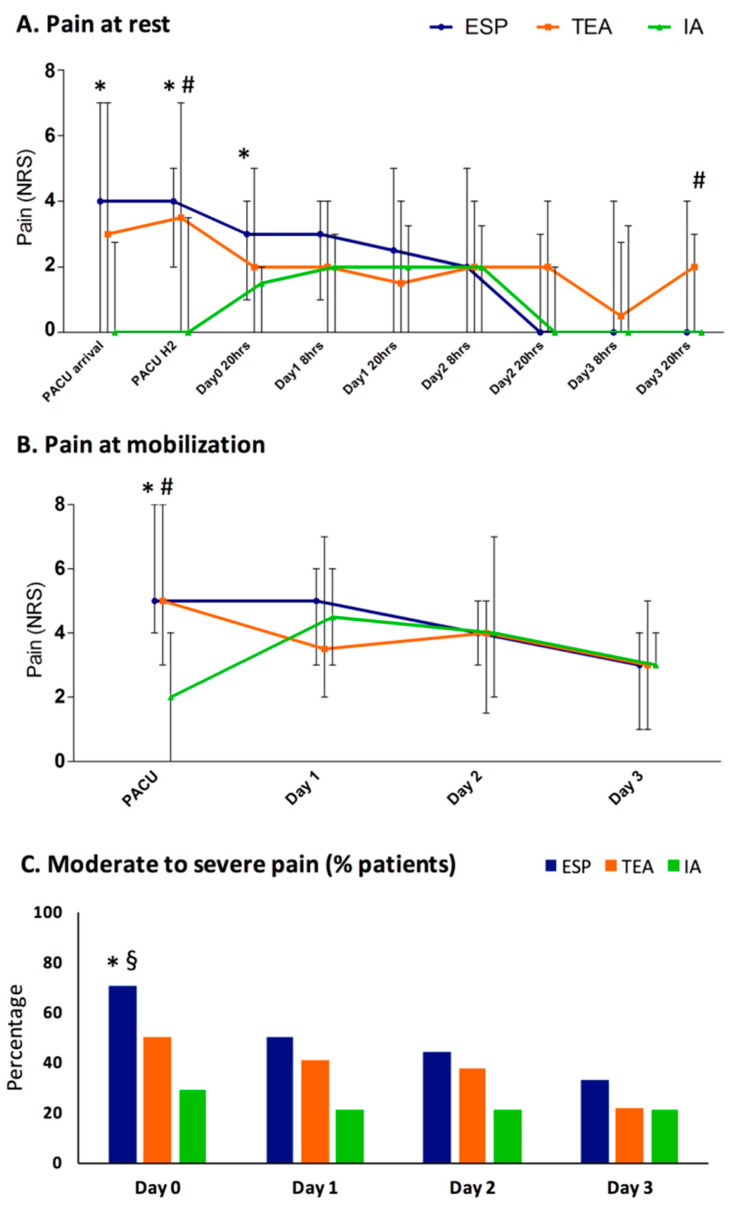
Postoperative pain in the three groups: (**A**) pain at rest; (**B**) pain during mobilization (during chest physiotherapy for Days 1 to 3); (**C**) percentage of patients with a moderate-to-severe pain (NRS ≥ 4). Points with blue line, ESP group; squares with orange line, TEA group; triangle with green line, IA group. The charts A and B show medians and (25–75%) interquartile range, and the chart C shows percentages. *, *p* < 0.05 between ESP and IA groups; #, *p* < 0.05 between TEA and IA groups; §, *p* < 0.05 between ESP and TEA groups. ESP, erector spinae plane block; IA, intrathecal analgesia; TEA, thoracic epidural analgesia; NRS, numerical rating scale of the pain; PACU, post-anesthesia care unit.

Detailed results of the pain scores measured between Day 0 and Day 3 are presented in Figure 2. In the post-anesthesia care unit (PACU), pain was lower in the IA group than in other groups. There were no significant differences at rest on Days 1 and 2 between the three groups (Figure 2A). On H2 and on the evening of Day 3, pain at rest was significantly lower in the IA group, in comparison with the TEA group. During chest physiotherapy exercises, there was no significant difference in pain intensity between groups (Figure 2B). On Day 0, there was a significant higher rate of patients with at least one episode of moderate-to-severe pain (NRS ≥ 4) in the ESP group (Figure 2C).

All patients received opioids in the postoperative period, in the form of IV morphine for 21 (16%) patients, IV oxycodone for 96 (83%) patients, oral morphine for 1 (<1%) patients, and/or oral oxycodone for 87 (75%) patients. The details of opioids consumption are shown in Table 3. There was no significant difference in the rate of patients who required opioids throughout the hospitalization between the groups, except on H2 where a lower rate of patients required opioids in the TEA group than in the ESP group. The amounts of morphine equivalents received (oral or IV) were not significantly different between the groups on Days 0, 1 and 2 and in total until the third postoperative day. However, these amounts were higher on Day 3 in the TEA group.

All patients (except one deceased) had a postoperative consultation with the surgeon and/or the referring pulmonologist, and the presence of neuropathic pain was assessed for 83 (72%) of them (50 days after surgery on average). In these patients, the incidence of neuropathic pain was 34% and was not significantly different between the three groups, although higher in TEA and IA groups (14 (28%), 9 (41%) and 5 (45%) in ESP, TEA and IA groups, respectively, overall *p*-value = 0.39).

### 3.4. Postoperative Recovery

The first standing mobilization occurred earlier in the ESP group, and there were no significant differences in peak expiratory flows measured on Days 1, 2 and 3 (Table 4).

Lengths of ICU and hospital stays were significantly longer in the TEA group than in the two other groups (Table 4). When patients were hospitalized in ICU after the surgery, the length of stay in ICU was significantly shorter in the ESP group, in comparison with other groups.

There were significantly more lung infections, confusion, hypotension, supraventricular tachycardia and postoperative ileus in the TEA group compared to the ESP group. There were no significant differences between the three groups in other respiratory, hemodynamic, neurological, digestive, cardiac and urological complications (Supplementary Material, Table S1). Moreover, there were no significant differences in the rates of ICU readmission, early surgical revision or death.

### 3.5. Post Hoc Analysis in Patients Who Underwent a Thoracotomy

Forty-two patients underwent an elective or unplanned thoracotomy; 27 (64%) of them had a TEA and 9 (21%) an IA. In these patients, the length of ICU stay was reduced in those who had an IA in comparison with a TEA (2 (1–4) vs. 4 (3–6) days, respectively, *p*-value = 0.01). There were no significant differences in other analgesia and postoperative recovery parameters, except for pain at rest, which was significantly lower in the IA group on Day 3 in the evening (Supplementary Material, Table S2).

## 4. Discussion

In our center, a regional anesthesia strategy based on the surgical approach and on the risk of conversion to thoracotomy allowed for effective postoperative analgesia, regardless of the type of block used, and rapid recovery according to the type of surgery received. Interestingly, in the subgroup of patients who underwent a thoracotomy, postoperative pain and recovery tended to be better in patients who received intrathecal analgesia in comparison with epidural analgesia.

Postoperative pain after lung surgery is often severe, and RA has shown its value in this context. Epidural analgesia has long been shown to be the gold standard for this surgery because of its benefits for postoperative pain, pulmonary function and on limiting the side effects associated with the use of morphine [26,27]. However, two important developments marked a turning point for pain and recovery after thoracic surgery: the development of VATS [28], which limits the surgical incision and prevents pain associated with rib fractures, and the development of lateral and posterior wall blocks, which limit the hemodynamic and respiratory repercussions linked to central blocks [29,30]. These blocks have been the subject of a relatively large number of studies, and most of them were shown to be effective, compared to a placebo [31,32] or epidural or other blocks [33]. In clinical practice, however, the choice of a technique remains difficult and often depends on the experience of the center and especially of the anesthesiologist in charge of the patient. In our center, we decided to adapt this choice to the risk of conversion to thoracotomy. To our knowledge, this type of strategy has been little evaluated, and, in particular, the three blocks that we chose have not been compared together, although they are likely to be frequently used in other centers.

We observed here that the use of these three blocks allowed adequate analgesia in patients, with less than 50% of patients who had at least moderate pain, and relatively low pain scores at rest. These results are compatible with those found in the existing literature. Indeed, with VATS, the mean pain score was most of the time inferior to 3 [34]. For thoracotomy, when multimodal analgesia included an RA, pain was low or moderate in most cases [14,28]. Furthermore, even if there was no intrathecal infusion when IA was used in our unit, there was no significant difference in pain scores from Day 0 to Day 2 between IA and TEA. Despite the small number of patients who had intrathecal analgesia in our study, this result is interesting because it confirms the effectiveness of this technique in thoracic surgery [35].

Despite the effectiveness of central blocks, more peripheral blocks such as pectoralis block, serratus block and ESP were proposed with the aim of facilitating recovery [36]. One of the underlying ideas is that, at the doses achieved, central blocks most often require hospitalization with continuous monitoring or hospitalization in ICU, which could slow down recovery, despite the less-invasive nature of the surgery (in the case of VATS in particular). In our study, as expected, patients who had an ESP were most of the time patients who had a VATS (not converted to thoracotomy) and we confirmed that their hospital length of stay was shorter than in the other groups. Interestingly, the inclusion of both VATS and thoracotomies in the same center allowed us to observe that patients who had a VATS experienced the most pain at Day 0 (vs. patients who had thoracotomy), despite a lower number of drains. This observation calls into question the use of ESP in this indication in comparison with other blocks such as the paravertebral block currently recommended in this indication [17]. In addition, when we compared TEA and IA groups, patients who had an IA had less severe pain on Day 3, but also a shorter ICU length of stay, including in the subgroup of patients who only had a thoracotomy. While studies have already compared IA with TEA in thoracic surgery [37], new, prospective and randomized controlled studies now seem necessary to verify whether intrathecal analgesia could allow an earlier recovery than epidural analgesia, especially since we know that early recovery can improve postoperative morbidity in this context.

Additionally, and interestingly, we did not observe any differences between IA and TEA using respiratory recovery criteria such as pain during chest physiotherapy, removal of drains, and early patient mobilization. On the other hand, this strategy made it possible, as desired, to obtain good results in patients who had an ESP, i.e., those who mainly had a VATS surgery, as already observed in previous studies [38,39].

Our study has some limitations, linked in part to its observational nature and the relatively low number of patients (number of patients over one year in our center). This design had the advantage of assessing in real practice the impact of our protocol on pain and recovery. Its limited duration made it possible to observe these results with similar anesthetic and surgical techniques, same anesthesiologists and surgeons, and with similar postoperative pain and rehabilitation managements. Moreover, the study included all types of surgical approaches, i.e., VATS, VATS converted to thoracotomies, and thoracotomies. This design resulted in the inclusion of patients with different postoperative conditions and an uneven number of patients between groups. However, this imbalance between the groups is likely consistent with the proportions of patients with a VATS or a thoracotomy in centers performing pulmonary resections. In addition, this choice allowed us to verify under similar conditions if this strategy, which included three possible RA techniques, was relevant for the management of all of these patients who were to undergo lung resection in our department. These results now encourage an exploration of the value of this strategy, or of strategies using other blocks, in controlled and multicentric trials.

## 5. Conclusions

In our institution, a regional anesthesia strategy based on the risk of conversion to thoracotomy—combining an erector spinae plane block for video-assisted thoracoscopic surgery, thoracic epidural analgesia for thoracotomy, and intrathecal analgesia as a possible alternative—allowed for adequate postoperative analgesia and rapid recovery. Interestingly, in the subgroup of patients who underwent a thoracotomy, some of these parameters were better in patients who received intrathecal analgesia, in comparison with epidural analgesia. These results need to be confirmed by stronger prospective and controlled studies.

## Figures and Tables

**Table 1:** Preoperative characteristics.

	All Patients (*n* = 116)	ESP Group (*n* = 70)	TEA Group (*n* = 32)	IA Group (*n* = 14)	*p*
**Patients’ characteristics**					
Age (years)	64 (11)	64 (11)	63 (10)	66 (12)	0.48
Male	73 (63%)	41 (59%)	21 (66%)	11 (79%)	0.32
Height (cm)	169 (9)	169 (9)	169 (8)	170 (10)	0.76
Weight (kg)	72 (17)	70 (16)	72 (18)	81 (22)	0.16
ASA status					
I	5 (4%)	2 (3%)	2 (6%)	1 (7%)	0.64
II	47 (41%)	28 (40%)	14 (44%)	5 (36%)	0.87
III	63 (54%)	39 (56%)	16 (50%)	8 (57%)	0.84
IV	1 (9%)	1 (1%)	0	0	0.72
**Medical history**					
COPD	39 (34%)	19 (27%)	16 (50%)	4 (29%)	0.08
Active smokers	27 (23%)	16 (23%)	7 (22%)	4 (29%)	0.88
Previous thoracic surgery	24 (21%)	13 (19%)	8 (25%)	3 (21%)	0.76
Previous thoracic radiotherapy	10 (9%)	6 (9%)	3 (9%)	1 (7%)	0.97
Diabetes	18 (16%)	12 (17%)	5 (16%)	1 (7%)	0.59
Chronic alcoholism	15 (13%)	10 (14%)	4 (12%)	1 (7%)	0.74
Psychiatric disease	8 (7%)	4 (6%)	3 (9%)	1 (7%)	0.80
Chronic pain	24 (21%)	16 (23%)	5 (16%)	3 (21%)	0.69
**Preoperative respiratory function**					
FEV1 (L)	2.4 (1.9–2.9)	2.5 (1.9–3.0)	2.4 (1.8–2.8)	2.0 (1.8–3.1)	0.36
Tiffeneau index	71 (61–79)	71 (63–80)	66 (57–78)	69 (60–78)	0.28
**Surgical indication**					
Diagnostic biopsy	5 (4%)	4 (6%)	0	1 (7%)	0.36
Lung infection	2 (2%)	1 (1%)	0	1 (7%)	0.22
Tumour resection	107 (92%)	65 (93%)	30 (93%)	12 (86%)	0.62
Other	2 (2%)	0	2 (6%)	0	0.07

Values are expressed as numbers (%), mean (standard deviation) or median (IQ 25–75%): ESP, erector spinae plane block; IA, intrathecal analgesia; TEA, thoracic epidural analgesia.

**Table 2:** Intraoperative anesthetic and surgical data.

	All Patients (*n* = 116)	ESP Group (*n* = 70)	TEA Group (*n* = 32)	IA Group (*n* = 14)	ESP vs. TEA *p* Value	ESP vs. IA *p* Value	TEA vs. IA *p* Value
**Intravenous anesthesia**							
Remifentanil (mg)	1.6 (1.0–2.0)	1.5 (0.9–1.9)	1.8 (1.4–2.6)	2.0 (1.5–2.6)	<0.01	0.01	0.74
Propofol (g)	1.7 (1.4–2.4)	1.6 (1.3–2.3)	1.9 (1.5–2.5)	2.2 (1.6–3.2)	0.07	0.01	0.24
**IV morphine equivalent (mg)**	4 (4–5)	4 (4–5)	4 (4–5)	5 (3.75–5.25)	0.57	0.9	0.69
Paracetamol	113 (97%)	69 (98%)	30 (94%)	14 (100%)	0.23	1	1
Nefopam	70 (60%)	43 (61%)	19 (59%)	8 (57%)	1	0.77	1
NSAIDs	22 (19%)	18 (26%)	2 (6%)	2 (14%)	0.03	0.5	0.57
Ketamine	78 (67%)	52 (74%)	23 (72%)	3 (21%)	0.81	<0.01	<0.01
**Surgical incision**							
Primary thoracotomy	38 (33%)	3 (4%)	27 (84%)	8 (57%)	<0.01	<0.01	0.24
Converted VATS	8 (7%)	3 (4%)	0	1 (%)	0.55	0.52	0.3
Not converted VATS	70 (60%)	64 (91%)	5 (16%)	5 (%)	<0.01	<0.01	0.24
**Type of resection**							
Lobectomy	69 (59%)	37 (53%)	23 (72%)	9 (64%)	<0.01	0.24	0.17
Pneumonectomy	7 (6%)	0	6 (19%)	1 (7%)	<0.01	0.16	0.65
Segmentectomy	7 (6%)	7 (10%)	0	0	0.33	1	0.41
Wedge	32 (28%)	26 (37%)	2 (6%)	4 (29%)	0.26	1	0.49
Biopsy	1 (8%)	0	1 (3%)	0	<0.01	0.02	0.4
**Curage**	80 (69%)	43 (61%)	26 (81%)	11 (79%)	0.06	0.3	1
**Drains**	1 (1–2)	1 (1–1)	2 (1–2)	1.5 (1–2)	<0.01	0.01	0.57
**Rib fractures**	4 (3%)	1 (1%)	3 (9%)	0	0.09	1	0.54
**Operating room time (min)**	173 (134–210)	170 (129–204)	191 (143–238)	170 (150–219)	0.03	0.44	0.42
**PACU time (min)**	131 (114–169)	132 (120–165)	120 (84–256)	131 (67–195)	0.39	0.7	0.88

Values are expressed as numbers (%) or median (IQ 25–75%): ESP, erector spinae plane block; IA, intrathecal analgesia; NSAIDs, non-steroidal anti-inflammatory drugs; PACU, post-anesthesia care unit; TEA, thoracic epidural analgesia; VATS, video-assisted thoracoscopic surgery.

**Table 3:** Postoperative pain and opioid consumption.

	All Patients (*n* = 116)	ESP Group (*n* = 70)	TEA Group (*n* = 32)	IA Group (*n* = 14)	ESP vs. TEA *p* Value	ESP vs. IA *p* Value	TEA vs. IA *p* Value
**IV or oral opioid use**							
Day 0	115 (99%)	70 (100%)	31 (97%)	14 (100%)	0.31	1	1
Day 1	95 (82%)	64 (91%)	19 (59%)	12 (86%)	<0.01	0.61	0.09
Day 2	84 (72%)	50 (71%)	24 (75%)	10 (71%)	0.81	1	1.0
Day 3	69 (59%)	38 (54%)	23 (71%)	8 (57%)	0.12	1	0.49
**IV morphine equivalent (for oral or IV opioids) (mg)**							
H2	3 (0–7)	5 (0–8)	0 (0–3.5)	0 (0–4.5)	<0.01	0.03	0.97
Day 0	14 (5–25)	16 (7–25)	10 (0–27)	16 (5–33)	0.16	0.94	0.46
Day 1	12.3 (2.5–23.3)	12 (6.5–22)	6.5 (0–35)	16 (6–24.5)	0.31	0.6	0.49
Day 2	10 (0–14.1)	8.8 (0–12.5)	11 (0.3–29.5)	7.5 (0–16.3)	0.05	0.69	0.28
Day 3	5 (0–10)	5 (0–10)	10 (0–18)	2.75 (0–10)	<0.01	0.9	0.049
Total	44 (28.9–73.3)	44 (28.9–73.3)	41 (18–94.9)	57 (19.1–77.1)	0.96	0.80	0.86

Values are expressed as numbers (%) or median (IQ 25–75%). ESP, erector spinae plane block; IA, intrathecal analgesia; TEA, thoracic epidural analgesia.

**Table 4:** Postoperative recovery parameters.

	All Patients (*n* = 116)	ESP Group (*n* = 70)	TEA Group (*n* = 32)	IA Group (*n* = 14)	ESP vs. TEA *p* Value	ESP vs. IA *p* Value	TEA vs. IA *p* Value
**First time setting in the chair (days)**	1 (1–1)	1 (1–1)	1 (1–1)	1 (1–1)	0.02	0.18	0.49
**First standing up (days)**	1 (1–1)	1 (1–1)	1 (1–2)	1 (1–2)	<0.01	0.01	0.94
**Drain removal ≤ Day 3**	68 59%)	52 (74%)	9 (28%)	7 (50%)	<0.01	0.1	0.18
**Urinary catheter removal ≤ Day 3**	76 (66%)	39 (93%)	25 (78%)	12 (92%)	0.09	1	0.4
**PEF (% theoretical value)**							
Day 1	35 (25–46)	37 (27–47)	35 (27–43)	25 (21–47)	0.62	0.37	0.44
Day 2	30 (24–38)	28 (24–40)	31 (21–35)	31 (24–38)	0.8	0.8	0.6
Day 3	37 (27–46)	41 (29–46)	31 (17–54)	34 (29–53)	0.19	0.7	0.42
**Postoperative admission to ICU** **(vs. ward)**	93 (80%)	47 (67%)	32 (100%)	14 (100%)	<0.01	<0.01	1
**Length of ICU stay (days)**	2 (1–4)	1 (0–2)	4 (3–6)	1 (1–2.5)	<0.01	<0.01	<0.01
**Length of hospital stay (days)**	5 (3–9)	4 (3–5)	10 (6–15)	6 (4–11)	<0.01	0.07	0.046

Values are expressed as numbers (%) or median (IQ 25–75%). ESP, erector spinae plane block; IA, intrathecal analgesia; ICU, Intensive care unit; PEF, Peak expiratory flow; TEA, thoracic epidural analgesia.

## Data Availability

The data presented in this study are available on request from the corresponding author.

## Supplementary Materials

The following supporting information can be downloaded at: https://www.mdpi.com/article/10.3390/jcm11051376/s1, Figure S1: Flow chart of the study; Table S1: Post-operative complications; Table S2: Post hoc analysis in patients who underwent a thoracotomy.
