# Supplementary material for: Impact on Postoperative Pain and Recovery of a Regional Analgesia Strategy Based on the Surgical Approach for Lung Resection: A Prospective Observational Study
Source: J Clin Med. 2022 Mar 2;11(5):1376. doi: 10.3390/jcm11051376 (PMC8911238; doi:10.3390/jcm11051376)
Supplement: Supplementary file 1 [file jcm-11-01376-s001.zip › jcm-1587001-supplementary.pdf]

Supplementary Materials

Impact on Postoperative Pain and Recovery of a Regional Analgesia Strategy Based on the Surgical Approach for Lung Resection: A Prospective Observational Study

Marion Trouillard [1], William Dupuis [1], Hélène Siaudeau [1], Florian Denou [1], Emmanuelle Longeau [1], Maxime Léger [1], Myriam Ammi [2], Cyril Sargentini [1], Sigismond Lasocki [1] and Emmanuel Rineau [1,*]

[1] Department of Anesthesiology and Intensive Care, University Hospital of Angers, 49100 Angers, France; marion.trd@gmail.com (M.T.); williamj.dupuis@hotmail.fr (W.D.); helene.siaudeau@chu-angers.fr (H.S.); florian.denou@hotmail.fr (F.D.); emmanuelle.longeau@chu-angers.fr (E.L.); maxime.leger@chu-angers.fr (M.L.); cysargentini@chu-angers.fr (C.S.); silasocki@chu-angers.fr (S.L.)

[2] Department of Cardiovascular and Thoracic Surgery, University Hospital of Angers, 49100 Angers, France; myriam.ammi@chu-angers.fr

* Correspondence: emmanuel.rineau@chu-angers.fr; Tel.: +33-2-41-35-39-51

Figure S1. Flow chart of the study.

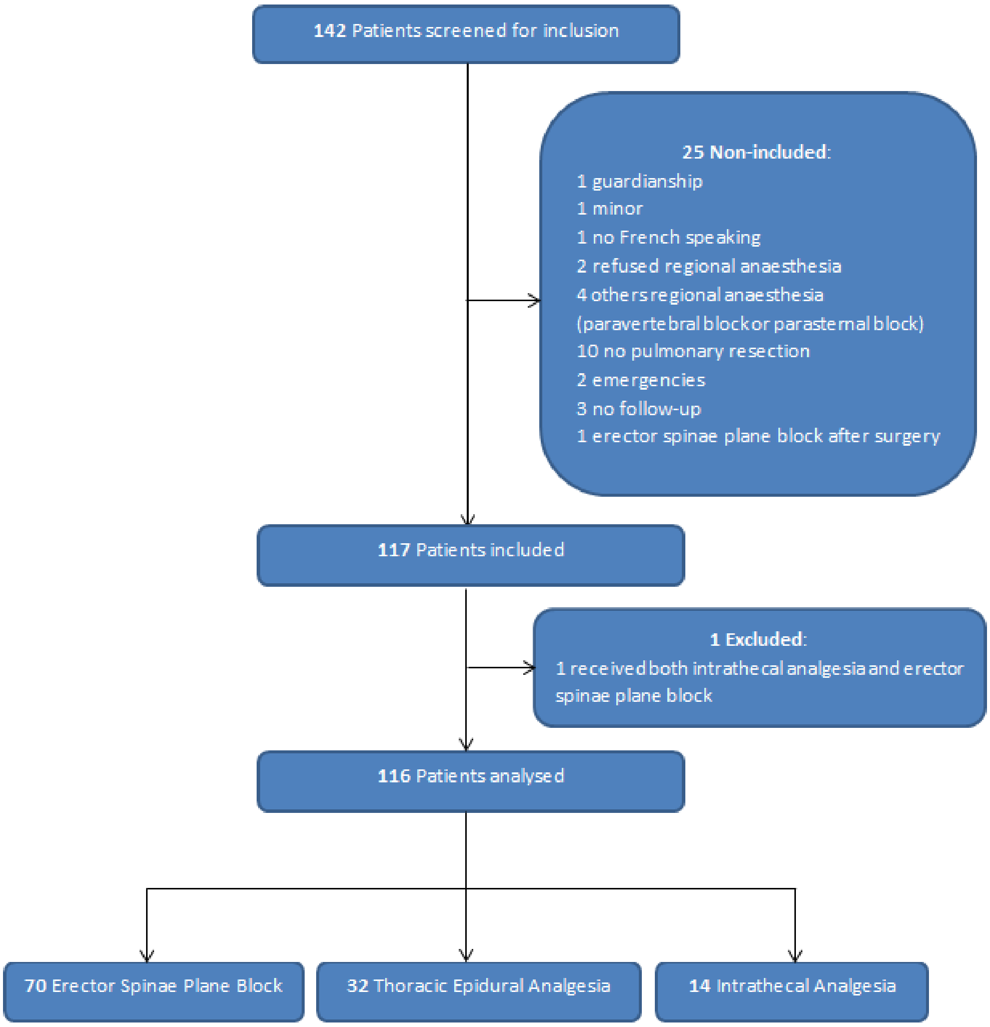

Table S1. Postopoperative complications.

	ESP Group (n = 70)	TEA Group (n = 32)	IA Group (n = 14)	ESP vs. TEA *p* Value	ESP vs. IA *p* Value	TEA vs. IA *p* Value
Lung infections	3 (4%)	7 (22%)	2 (14%)	0.01	0.19	0.7
Fibroscopic aspiration	4 (6%)	6 (19%)	2 (14%)	0.07	0.26	1
NIV	4 (6%)	5 (16%)	2 (14%)	0.13	0.26	1
High flow oxygen therapy	2 (3%)	3 (9%)	0	0.17	1	0.54
Re-intubation	1 (1.4%)	2 (6%)	1 (7%)	0.23	0.3	1
Thoracic drainage	2 (3%)	0	0	1	1	
Epidural haematoma	0	0	0			
Motor dysfunction	0	0	0			
Transient radicular irritation	0	0	0			
Confusion	1 (1.4%)	5 (16%)	0	0.01		0.3
Over sedation	0	0	1 (7%)		0.16	0.3
Hypotension	6 (9%)	11 (34%)	2 (14%)	<0.01	0.61	0.28
Supraventricular tachycardia	2 (3%)	7 (22%)	1 (7%)	<0.01	0.42	0.4
Cardiorespiratory arrest	0	1 (3%)	0	0.31		1
Acute heart failure	1 (1.4%)	0 (0)	0 (0)	1	1	
PONV	14 (20%)	10 (31%)	1 (7%)	0.2	0.44	0.13
Postoperative ileus	12 (17%)	13 (40%)	3 (21%)	0.01	0.7	0.31
Acute urinary retention	7 (10%)	3 (9%)	1 (7%)	1	1	1
ICU re-admission	0	0	1 (7%)		0.16	0.3
Revision surgery	1 (1.4%)	1 (3%)	1 (7%)	0.53	0.3	0.52
Death	0	1 (3%)	0	0.31		1
Neuropathic pain	14 (28%)	9 (41%)	5 (45%)	0.29	0.29	1

Values are expressed as numbers (%)

NIV, Non-invasive ventilation; PONV, Postoperative nausea and vomiting; ICU, Intensive care unit

Table S2. Post hoc analysis in patients who underwent a thoracotomy

	**TEA Group**	**IA Group**	***p* Value**
Sample size, n	27	9	
Length of ICU hospitalisation stay (days)	4 [3–6]	2 [1–4]	0.01
Length hospitalisation stay (days)	10 [7–15]	11 [6–12]	0.65
Total IV morphine equivalent (mg)	44 [12.5–97.5]	73 [52–89]	0.51
Pain on admission	3 [0–7.25]	0 [0–2.75]	0.22
Pain at H2	3 [1–6.5]	0.5 [0–5]	0.16
Pain at Day 0	2 [0–4]	2 [0.5–2.5]	0.73
Pain at rest in morning			
Day 1	2 [0–4]	2 [0–3.5]	0.52
Day 2	2 [0–3]	2 [0–4]	0.63
Day 3	0 [0–2]	3 [0–3.5]	0.28
Pain at rest in evening			
Day 1	2 [0–4]	2 [0–4]	0.79
Day 2	2 [0–3]	1 [0–2]	0.37
Day 3	2 [0–3]	0 [0–0]	0.03
Pain at chest physiotherapy			
Day 1	3 [2–7]	6 [3.5–7]	0.1
Day 2	4 [1–5]	7 [2–7]	0.21
Day 3	3 [1–5]	3 [3–4]	0.25
Lung infections	6 (22%)	2 (22%)	1
Fibroscopic aspiration	5 (18.5%)	2 (22%)	1
NIV	5 (18.5%)	2 (22%)	1
High flow oxygen therapy	3 (11%)	0	0.55
Re-intubation	2 (7%)	1 (11%)	1
Confusion	5 (18.5%)	0	0.3
Hypotension	11 (41%)	2 (22%)	0.44
Supraventricular tachycardia	7 (26%)	1 (11%)	0.64
Cardiorespiratory arrest	1 (4%)	0	1
PONV	6 (22%)	0	0.3
Postoperative ileus	10 (37%)	3 (33%)	1
Acute urinary retention	3 (11%)	0	0.55
ICU re-admission	0	1 (11%)	0.25
Revision surgery	1 (4%)	1 (11%)	0.44
Death	1 (4%)	0	1
Neuropathic pain	6 (35%)	4 (50%)	0.48
First time setting in the chair (days)	1 [1–1]	1 [1–1]	0.37
First standing up (days)	1 [1–2]	1 [1–1]	0.77
Drain removal ≤ Day 3	8 (30%)	6 (22%)	1
Urinary catheter removal ≤ Day 3	20 (74%)	12 (89%)	0.65

Values are expressed as numbers (%) or median [interquartiles 25–75%]

NIV, Non-invasive ventilation; PONV, Postoperative nausea and vomiting; ICU, Intensive care unit
